# Metabolism-regulated ferroptosis in cancer progression and therapy

**DOI:** 10.1038/s41419-024-06584-y

**Published:** 2024-03-08

**Authors:** Lvlan Ye, Xiangqiong Wen, Jiale Qin, Xiang Zhang, Youpeng Wang, Ziyang Wang, Ti Zhou, Yuqin Di, Weiling He

**Affiliations:** 1 Department of Gastrointestinal Surgery, The First Affiliated Hospital, Sun Yat-sen University, Guangzhou, Guangdong 510080 China; 2 Department of Gastrointestinal Surgery, Xiang’an Hospital of Xiamen University, School of Medicine, Xiamen University, Xiamen, Fujian 361000 China; 3 Department of Biochemistry, Zhongshan School of Medicine, Sun Yat-sen University, Guangzhou, Guangdong 510080 China; 4 Center for Translational Medicine, The First Affiliated Hospital, Sun Yat-sen University, Guangzhou, Guangdong 510080 China; 5 Molecular Diagnosis and Gene Testing Center, The First Affiliated Hospital, Sun Yat-sen University, Guangzhou, Guangdong 510080 China; 6 Department of Liver Surgery, The First Affiliated Hospital of Sun Yat-Sen University, Guangzhou, Guangdong 510080 China

**Keywords:** Cell death, Cancer metabolism, Cell signalling

## Abstract

Cancer metabolism mainly includes carbohydrate, amino acid and lipid metabolism, each of which can be reprogrammed. These processes interact with each other to adapt to the complicated microenvironment. Ferroptosis is a regulated cell death induced by iron-dependent lipid peroxidation, which is morphologically different from apoptosis, necrosis, necroptosis, pyroptosis, autophagy-dependent cell death and cuprotosis. Cancer metabolism plays opposite roles in ferroptosis. On the one hand, carbohydrate metabolism can produce NADPH to maintain GPX4 and FSP1 function, and amino acid metabolism can provide substrates for synthesizing GPX4; on the other hand, lipid metabolism might synthesize PUFAs to trigger ferroptosis. The mechanisms through which cancer metabolism affects ferroptosis have been investigated extensively for a long time; however, some mechanisms have not yet been elucidated. In this review, we summarize the interaction between cancer metabolism and ferroptosis. Importantly, we were most concerned with how these targets can be utilized in cancer therapy.

## FACTS

- Although the mechanism of metabolic enzymes or metabolites on ferroptosis has been studied extensively in cancer cells, there are still many mysteries that need to be clarified.
- It is well known that ferroptosis can be triggered by phospholipid peroxidation. However, the specific executor of ferroptosis remains unclear.
- The effect of immunotherapy is poor in some cancer therapy. The combination of immunotherapy and ferroptosis-targeted therapy might improve cancer treatment.

## OPEN QUESTIONS

- Why do some molecules play opposite roles in ferroptosis, and what is the underlying mechanisms?
- Which molecule acts as an executor in ferroptosis?
- Which molecules can be utilized to combine immunotherapy and ferroptosis-targeted therapy in cancer treatment?

## Introduction

In the process of cellular life, cell death is inevitable and can be caused by various factors, including intracellular and extracellular stimuli such as genes, drugs and other environmental factors. Cell death is classified into regulated and accidental cell death based on the rate at which it occurs and the degree of impact of drugs and genes [1]. Based on the different morphological, biochemical, and functional characteristics, regulated cell death is subdivided into intrinsic apoptosis, extrinsic apoptosis, mitochondrial permeability transition (MPT)-driven necrosis, necroptosis, ferroptosis, pyroptosis, parthanatos, entotic cell death, NETotic cell death, lysosome-dependent cell death, autophagy-dependent cell death, immunogenic cell death, mitotic catastrophe and cuprotosis [1–3]. Ferroptosis is a new term introduced by Dixon et al. [4] to describe non-apoptotic programmed cell death initiated by the overwhelming accretion of lethal lipid peroxides depending on the accumulation of intracellular iron [4]. The basic mechanism of ferroptotic death is that the peroxidation of polyunsaturated fatty acids (PUFAs) on the cellular membrane is mainly catalyzed by lipoxygenase, through an enzymatic pathway, or ferrous ions, through non-enzymatic autoxidation propelled by the Fenton reaction [5]. Morphologically, the features of ferroptosis are mainly observed in mitochondria, including shrinkage of mitochondria, higher density of the outer mitochondrial membrane and reduced or absent mitochondrial cristae.

The term “metabolism” is derived from the Greek word “change”, which refers to a series of orderly chemical reactions for life maintenance that occur in cells, tissues or organisms. Metabolism includes material metabolism and energy metabolism; the former refers to the ingestion of nutrients necessary for life activities and the discharge of metabolic waste harmful for life, and the latter refers to the exchange of energy between organisms and the external environment or the transformation of energy within cells, tissues and organisms. Cellular material metabolism primarily consists of carbohydrate, amino acid and lipid metabolism. In 1924, Otto Warburg observed a curious phenomenon that, unlike other normal cells in vivo, cancer cells prefer glycolysis rather than aerobic respiration even under the condition that the oxygen is sufficient [6]. Since then, this phenomenon has been known as the Warburg Effect, and our understanding of cancer metabolism has been gradually deepened [6, 7]. Anabolism, catabolism and energy metabolism in cancer cells are reprogrammed to facilitate their proliferation and metastasis [8, 9]. In particular, carbohydrate, amino acid and lipid metabolism have been significantly modified in tumor cells [8, 10]. These three types of metabolism interact with each other and cooperate to support tumor survival and progression [10, 11].

The occurrence of ferroptosis is regulated by multiple positive and negative factors. PUFAs, iron and reactive oxygen species (ROS) are the main positive factors that are essential for triggering ferroptosis. PUFAs can be converted to PUFA-containing phospholipids (PUFA-PLs) by ACSL4 and LPCAT3, which then react with ROS utilizing iron as a catalyst, thus triggering lipid peroxidation and ferroptosis [5, 12, 13]. In addition to non-enzymatic processes, PUFA-PL peroxidation can be initiated enzymatically through the catalysis of cytochrome P450 oxidoreductase (POR) or arachidonate lipoxygenase (ALOX) [14]. Additionally, recent studies have identified three antioxidants that mainly suppress ferroptosis: (1) Reduced glutathione (GSH) is a canonical antioxidant in vivo, which can be used by glutathione peroxidase 4 (GPX4) to defend against ferroptosis [15]. (2) Ubiquinol (CoQH2) can be utilized to impede ferroptosis via different pathways. In the plasma membrane, CoQH2 can be produced from ubiquinone (oxidized CoQ) by ferroptosis suppressor protein 1 (FSP1) [16]. In the inner mitochondrial membrane, CoQH2 can be reduced from CoQ by dihydroorotate dehydrogenase (DHODH) to scavenge lipid peroxides and inhibit ferroptosis [17]. (3) Tetrahydrobiopterin (BH4) biosynthesis is catalyzed by a series of enzymes, among which GTP cyclohydrolase 1 (GCH1) is the rate-limiting enzyme [18]. BH4 acts as a lipid peroxyl radical trap, which can downregulate the levels of lipid peroxidation and thus defend against ferroptosis [19].

Of note, oxygen and iron are essential for cancer cell metabolism, whereas it is inevitable to generate ROS in these processes; therefore, ferroptosis is more likely a byproduct of metabolism [20]. Although the mechanism of metabolic enzymes or metabolites on ferroptosis has been studied extensively in cancer cells, there are still many mysteries that need to be solved. In this review, we summarize the interplay between ferroptosis and cell metabolism in cancer and discuss novel targets for cancer therapy.

## Ferroptosis and carbohydrate metabolism

**Fig. 1:**
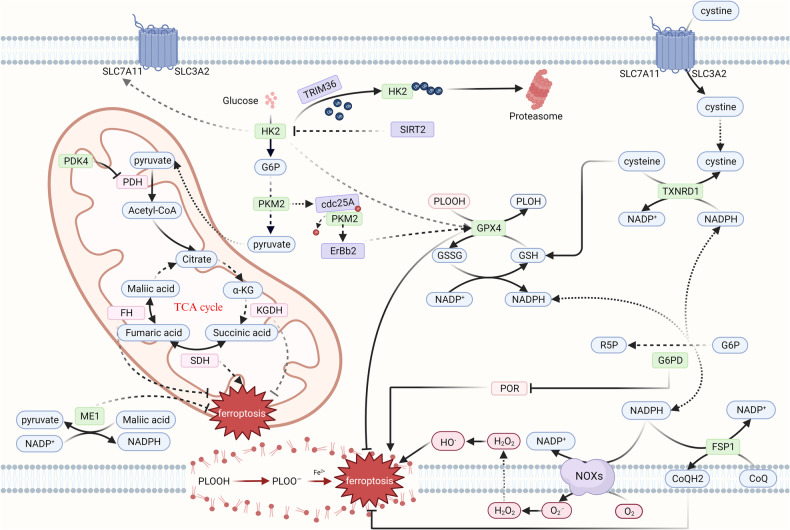
Ferroptosis and carbohydrate metabolism. Carbohydrate metabolism mainly contain glycolysis, TCA cycle and pentose phosphate pathway. The substrates and enzymes in these pathways affect ferroptosis in various mechanism. SLC7A11 solute carrier family 7 member 11, SLC3A2 solute carrier family 3 member 2, HK2 hexokinase 2, SIRT2 sirtuin 2, TRIM36 trigred motif 36, G6P glucose-6-phosphate, PKM2 pyruvate kinase M2, Cdc25 cell division cycle 25, GPX4 glutathione peroxidase 4, PLOOH phospholipid hydroperoxides, PLOH phospholipid alcohols, GSH reduced glutathione, GSSG glutathione disulfide, NADPH reduced nicotinamide adenine dinucleotide phosphate, PDK4 pyruvate dehydrogenase kinase 4, PDH pyruvate dehydrogenase complex, αKG alpha-ketoglutaric acid, KGDH alpha-ketoglutarate dehydrogenase complex, SDH succinate dehydrogenase, FH fumarate hydratase, TCA cycle tricarboxylic acid cycle, ME1 malic enzyme 1, TXNRD1 thioredoxin reductase 1, G6P glucose 6-phosphate, R5P ribose 5-phosphate, G6PD glucose-6-phosphate dehydrogenase, POR cytochrome p450 oxidoreductase, FSP1 ferroptosis suppressor protein 1, CoQ ubiquinone, CoQH_2_ ubiquinol, NOXs NADPH oxidases, O_2_ oxygen, O2^.−^ superoxide anion, H_2_O_2_ hydrogen peroxide, HO^.^ hydroxyl ion (Created with BioRender.com).

Glucose, is a kind of essential nutrient for organisms, which provides energy and carbon for maintaining life activities. In addition to functioning as an energy provider, glucose can also be transformed to other carbon containing compounds. Therefore, carbohydrate metabolism, is a hub of different kinds of material metabolism, which can be divided into glycolysis, aerobic oxidation, the pentose phosphate pathway (PPP), the uronic acid pathway, the polyol pathway, glycogen synthesis and glycogenolysis, gluconeogenesis, etc. Among the metabolic pathway mentioned above, glycolysis, the tricarboxylic acid cycle (TCA cycle) and the PPP are the research hotspots in the field of carbohydrate metabolism and ferroptosis in tumors (Fig. 1).

### Glycolysis

In recent years, researchers have found that ferroptosis can regulate glycolysis and that glycolysis can influence ferroptosis. For instance, some glycolytic enzymes or metabolic intermediates might directly intervene in ferroptosis. In addition, some proteins can stimulate tumor cells to switch from oxidative phosphorylation (OXPHOS) to glycolysis, in turn leading to lower levels of GSH, which suppress the activity of GPX4 and boost the accumulation of lipids, thus resulting in ferroptosis [21–24]. Hexokinase, phosphofructokinase-1 and pyruvate kinase are crucial glycolytic enzymes limiting the rate of glycolysis. Some researchers have found that some proteins can downregulate the expression of glycolysis-related protein hexokinase 2 (HK2) to induce ferroptosis through specific pathways [25, 26]. In the following study by Zhao et al., it has been identified that HK2 can be degraded by tripartite motif containing 36 (TRIM36) through ubiquitination, which leads to the reduction of GPX4 and facilitates ferroptosis in tumor cells [21]. Meanwhile, the glycolysis-related LINC02432/Hsa-miR-98–5p/HK2 axis restrains ferroptosis, which might upregulate the expression of solute carrier family 7 member 11 (SLC7A11) [27]. Taken together, these results show that HK2 might downregulate ferroptosis through activating the activity of GPX4 or facilitating the expression of SLC7A11. Pyruvate kinase M2 (PKM2), an isoform of pyruvate kinase, plays an important role in glycolysis in tumors and also participates in tumorigenesis [28, 29]. In order to elucidate the role of PKM2 in ferroptosis, He et al. discovered that PKM2 is dephosphorylated by cdc25A in the nucleus to inhibit ferroptosis induced by sorafenib [30]. Moreover, in a recent study conducted by Yin et al., they demonstrate that the interaction of PKM2 and voltage-dependent anion channel 3 (VDAC3) was disrupted by Compound 8, thus triggering ferroptosis [31, 32]. In line with these insights, it has been observed that PKM2 represses ferroptosis, evidence by increased cell viability and GPX4 expression and reduced levels of lipid ROS and ferritin heavy chain (FTH) [33]. Consistent with these findings, other researchers discovered that RSL3 can downregulate the levels of HK2, phosphofructokinase (PFKP) and PKM2, which makes glycolysis dysfunctional, thus enhancing the sensitivity of tumors to ferroptosis [34]. Moreover, in a study by Yu et al., glucose-6-phosphate isomerase (GPI), an enzyme converting glucose-6-phosphate to fructose-6-phosphate, was shown to be closely related to ferroptosis [35]. However, the specific mechanism of GPI in ferroptosis requires more researches to clarify.

Pyruvate oxidation is catalyzed by pyruvate dehydrogenase complex (PDH), which connects glycolysis and the TCA cycle. Once PDH is inhibited by pyruvate dehydrogenase kinase 4 (PDK4), pyruvate oxidation and the production of fatty acids are blocked, thus hampering ferroptosis and facilitating the development of cancers [36].

### TCA cycle

The TCA cycle is the common metabolic pathway for the breakdown of the three major nutrients, carbohydrate, lipids and amino acids. Through this cycle, acetyl-CoA can be oxidized completely and sufficient reducing equivalents can be generated for both the electron transfer chain and OXPHOS. Alpha-ketoglutarate dehydrogenase complex (KGDH), is a rate-limiting enzyme of the TCA cycle, which catalyze the formation of succinyl CoA from the oxidative decarboxylation of alpha-ketoglutarate. In a recent study by Roh et al., the authors confirm that gene silencing of dihydrolipoamide dehydrogenase (DLD), the E3 unit of the KGDH complex, represses ferroptosis caused by cystine deprivation or import inhibition [37]. Consistent with these insights, it has been reported that alpha-ketoglutaric acid (αKG), produced from isocitrate catalyzed by isocitrate dehydrogenase (IDH), and its downstream metabolites including succinic acid, fumaric acid, and malic acid, can displace the function of glutamine in the accumulation of lipid ROS and cystine starvation induced or system Xc^−^ inhibition induced ferroptosis [25, 38, 39]. In addition, a study by Tong et al. clarified the mechanism of succinate dehydrogenase (SDH) in ferroptosis. SDH, an enzyme in TCA cycle and consisting of Complex II in mitochondria respiratory chain, sensitizes cancer cells to ferroptosis through increased lipid peroxidation and ROS accumulation [40]. Furthermore, fumarate hydratase (FH), an enzyme that converts fumarate into malate in the TCA cycle, makes cancers more sensitive to ferroptosis induced by cystine depletion [23, 41]. Notably, a bioinformatics analysis by Li et al. demonstrated that the gene cluster of pyruvate metabolism and TCA cycle enzymes can forecast the potency of ferroptosis-induced therapy [42]. Remarkably, malic enzyme 1 (ME1), which yields pyruvate from malate and converts NADP^+^ to reduced nicotinamide adenine dinucleotide phosphate (NADPH), might reduce the accumulation of ROS and redox-active iron, hence resisting ferroptosis induced by inhibition of SLC7A11 [43]. Overall, it is implied that some enzymes or metabolites in the TCA cycle correlate with ferroptosis.

### Pentose phosphate pathway

The PPP is one of the glucose metabolism pathways parallel to glycolysis. PPP includes oxidative and non-oxidative phases, during which glucose 6-phosphate (G6P), the intermediate product in glycolysis, can be transformed into fructose-6-phosphate (F-6-P), glyceraldehyde-3-phosphate and ribose phosphate, and NADP^+^ can be reduced to NADPH [44–46]. Recently, mounting evidence has shown that NADPH plays an important role in ferroptosis. For instance, a report by Hayes et al. showed that NADPH provides hydrogen ions for cystine to generate cysteine, which might influence the production of GSH and the reduction of ROS, thereby suppressing ferroptosis [47]. Moreover, a study by Marcus Conrad et al. demonstrated that NADPH was used to regenerate CoQH_2_ by ferroptosis suppressor protein 1 (FSP1), which restrains ferroptosis [16]. Consistent with these findings, other studies confirmed that NADPH was used to generate NO, which suppressed ferroptosis [48, 49]. Otherwise, NADPH contributes to ferroptosis induced by NADPH oxidases (NOXs), which can provide one electron to oxygen to generate ROS [50]. As an electron donor, NADPH has the opposite functions. On the one hand, it can provide electrons to oxygen to produce hydrogen peroxide, thereby generating peroxidation of membrane phospholipids and then inducing ferroptosis [14]; on the other hand, NADPH donates electrons to reduce oxidized glutathione or CoQ, which upregulates the enzyme activity of GPX4 or FSP1 and inhibits lipid peroxidation, thus downregulating ferroptosis [16, 51]. In conclusion, these findings identify NADPH as a multiprong regulator of ferroptosis. Moreover, a great deal of evidence suggests that many enzymes regulate ferroptosis by adjusting NADPH flux [52, 53]. For example, lysine demethylase 5C (KDM5C), a histone demethylase, can regulate the expression of glucose-6-phosphate dehydrogenase (G-6-PD) primarily through its enzymatic activity [54]. G-6-PD, a rate-limiting enzyme in PPP, can control the flux of G6P and NADPH [55, 56]. As mentioned above, NADPH is a coenzyme of glutathione reductase, which catalyzes the formation of glutathione, thus counteracting ROS formation and ferroptosis in cancer cells [54]. Furthermore, a study by Yang et al. also find that G-6-PD suppresses ferroptosis via downregulating the expression of POR [57]. In conclusion, NADPH and G-6-PD in PPP act as suppressors in ferroptosis.

## Ferroptosis and amino acid metabolism

**Fig. 2:**
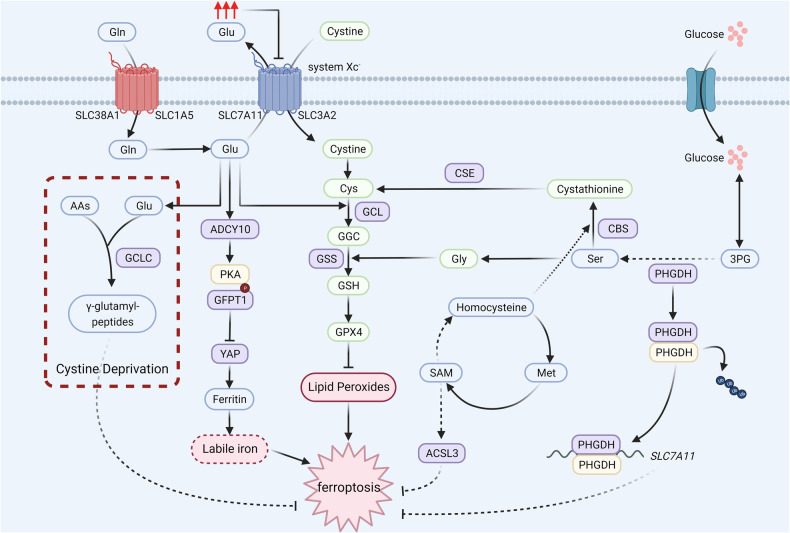
Ferroptosis and amino acid metabolism. Glutamate, cysteine and glycine are the composition of GSH, which is the negative regulator of ferroptosis. Intracellular glutamate is generated from glutamine. Cystine can be transported into cancer cells by system Xc^−^ and is reduced to cysteine. High levels of extracellular glutamate inhibit the activity of system Xc^−^. Besides, cysteine can be generated from serine, which also participate in the synthesis of glycine. The enzymes in amino acid metabolism suppress ferroptosis via regulating *SLC7A11*, labile iron and GPX4. Glu glutamate, Gln glutamine, SLC38A1 solute carrier family 38 member 1, SLC1A5 solute carrier family 1 member 5, SLC7A11 solute carrier family 7 member 11, SLC3A2 solute carrier family 3 member 2, AAs amino acids, ADCY10 adenylyl cyclase 10, PKA protein kinase A, GFPT1 glutamine-fructose-6-phosphate transaminase, YAP yes-associated protein, GCLC glutamate-cysteine ligase catalytic subunit, GCL glutamate-cysteine ligase, GSS glutathione synthetase, GSH reduced glutathione, GPX4 glutathione peroxidase 4, 3PG 3-phosphoglycerate, PHGDH phosphoglycerate dehydrogenase, CBS cystathionine beta-synthase, CSE cystathionine gamma-lyase, SAM S-adenosylmethionine, Met methionine, ACSL3 long-chain acyl-CoA synthetase 3 (Created with BioRender.com).

There are more than three hundred kinds of amino acids in nature, yet only twenty kinds of α-amino acids are involved in the synthesis of proteins [58]. Except serving as raw materials of proteins, amino acids play crucial roles in the formation of nitrogenous substances, including hormones, neurotransmitters and nucleotides. Studies have found that the ingestion and consumption of amino acids have been increased and the levels of the enzymes participating in amino acid anabolism and catabolism have been changed in cancer cells [59]. Furthermore, recent years have witnessed the important roles that amino acid metabolism play in ferroptosis (Fig. 2) [60].

### GSH

Among the 20 α-amino acids mentioned above, the function of glutamate, glycine, and cysteine metabolism in ferroptosis has attracted more attention. Glutamate, glycine, and cysteine can be combined to form glutathione, which can inhibit ferroptosis [61, 62]. High levels of extracellular glutamate can inhibit the transportation of cystine by system Xc^−^, resulting in an imbalance in antioxidative homeostasis and the occurrence of ferroptosis [4, 63]. Additionally, after inhibiting the activity of system Xc^−^, endogenous glutamate, become a decisive factor in the sensitivity of ferroptosis, which can regulate the ADCY/PKA/HBP/YAP axis in some tumor cells [64]. Cysteine, a non-essential amino acid, plays an important role in many diseases, such as cancer. It has been eloquently proved that cysteine deprivation decreases the synthesis of GSH and suppresses the enzymatic activity of GPX4, thus increasing lipid peroxidation and inducing ferroptosis [4, 65]. However, a recent study conducted by DeNicola discovered that the glutamate-cysteine ligase catalytic subunit (GCLC) can defend against ferroptosis induced by cysteine deprivation through facilitating the formation of γ-glutamyl-peptides, which can be enhanced by NRF2 [66]. Glycine and cysteine both originate from serine metabolism. Once serine is depleted, the synthesis of GSH is limited, which influences GPX4 stability and then induces ferroptosis [67]. Moreover, the first rate-limiting enzyme of the serine synthesis pathway, phosphoglycerate dehydrogenase (PHGDH), can couple with the RNA-binding protein poly (RC) binding protein 2 (PCBP2) and suppress its degradation, which plays a vital role in maintaining the stability of *SLC7A11* mRNA and upregulates its expression, ultimately obstructing the development of ferroptosis [68].

### Other amino acids

Methionine, an essential amino acid, can transform into S-adenosylmethionine (SAM), which can provide a methyl group for the synthesis of cysteine, thus stabilizing the activity of GPX4 and inhibiting ferroptosis [69, 70]. In addition, Yang et al. found methionine adenosyltransferase 2A (MAT2A), which participates in the methionine cycle, can synergize with ACSL3 to suppress ferroptosis by enhancing the promoter of ACSL3 [71]. Additionally, it has been found that cystathionine β-synthase (CBS), glutamic pyruvic transaminase 2 (GPT2) and suppressor of variegation 3-9 homolog 1 (SUV39H1) are related to amino acid metabolism and ferroptosis [72]. CBS, a rate-limiting enzyme in the transsulfuration pathway, catalyzes the irreversible transformation of homocysteine and serine to cystathionine, which is the precursor of cysteine [73, 74]. Increasing the enzyme activity of CBS can increase the synthesis of cysteine and GSH, thus reinforcing the function of GPX4, ultimately neutralizing lipid peroxides and suppressing ferroptosis [75]. In addition, Huang et al. demonstrated that CBS could function as a participant in resistance to ferroptosis induced by erastin, which is transcriptionally upregulated by NRF2 [76]. GPT2, a ferroptosis-related gene, catalyzes the reversible reaction between alanine and α-KG to generate pyruvate and glutamate [77, 78]. It is well established that GPT2 plays a key role in the development of cancer [77, 79–82]. However, the specific mechanism by which GPT2 regulates ferroptosis in cancer remains unclear. SUV39H1, a histone methyltransferase is known as a tumor suppressor, participates in the methylation of lysine 9 on Histone 3 [83]. The results of a study by Huang et al. provide new insights into the regulatory role of SUV39H1 in the progression of cancer, and show that SUV39H1 inhibits the transcription of dipeptidyl-peptidase-4 (DPP4) through catalyzing the methylation of DPP4 promoter, therefore reducing the production of lipid ROS and repressing ferroptosis [84, 85].

Branched-chain amino acid aminotransferase 2 (BCAT2), a key enzyme engaging in the metabolism of sulfur amino acids, can regulate the intracellular level of glutamate, which can maintain the function of system Xc^−^ and protect cancer cells from ferroptosis [86]. Tryptophan can be converted to indole-3-pyruvate (I3P) by interleukin-4-induced-1 (IL4i1), which protects cancer cells from ferroptosis through scavenging lipophilic radicals, increasing the GSH/ Glutathione disulfide (GSSG) ratio, upregulating the expression of SLC7A11 and targeting some genes in the antioxidant signaling pathway, such as hemeoxygenase-1 (HO-1) [87]. Together, these findings convincingly prove that amino acid metabolism has great effects on ferroptosis in cancer.

## Ferroptosis and lipid metabolism

**Fig. 3:**
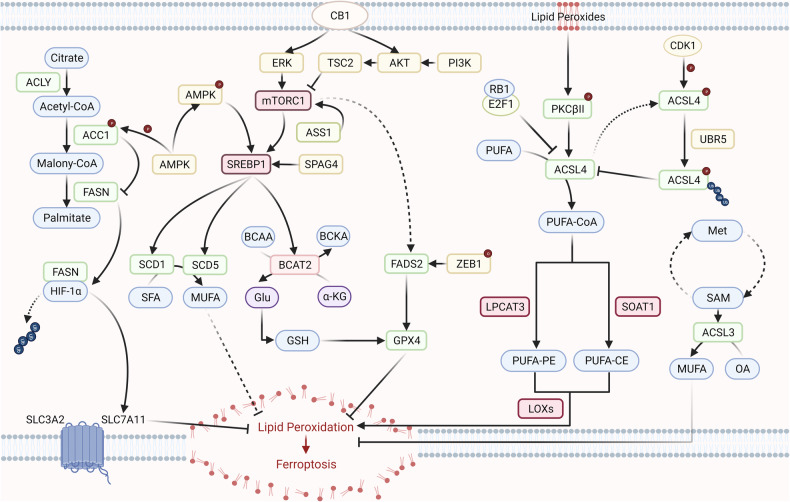
Ferroptosis and fatty acid metabolism. Fatty acid metabolism contains anabolism and catabolism. Fatty acid anabolism includes synthesis, elongation and desaturation. Citrate can be utilized to synthesize malony-CoA by ACLY and ACC1. ACC1 is phosphorylated and suppressed by AMPK, which inhibits the activity of FASN. FASN catalyzes the reaction of malony-CoA to palmitate, which can promote the deubiquitination of HIF-1α, thus increasing the expression of SLC7A11 and blocking ferroptosis. SREBP1 can regulate the activity of SCD1, SCD5 and BCAT2, which regulate ferroptosis through increasing the levels of MUFA and the activity of GPX4, respectively. ACSLs catalyze the decomposition of long-chain fatty acids. ACSL4, is regulated by RB1, PKCβII and CDK1, which catalyzes the formation of PUFA-CoA. PUFA-CoA can be converted to PUFA-PE or PUFA-CE by LPCAT3 and SOAT1, respectively. PUFA-PLs are used to produced lipid peroxides by LOXs, thus sensitize cancer cells to ferroptosis. ACSL3 is another member of ACSL family, which catalyzes the transformation of OA to MUFA, thus acting as a suppressor of ferroptosis. ACLY ATP citrate lyase; ACC1, acetyl-CoA-carboxylase 1, FASN fatty acid synthase, HIF-1α hypoxia-inducible factor-1α, SLC7A11 solute carrier family 7 member 11, SLC3A2 solute carrier family 3 member 2, CB1 cannabinoid receptor 1, ERK extracellular signal-regulated kinase, TSC2 tuberous sclerosis complex 2, AKT protein kinase B, PI3K phosphoinositide 3-kinase, mTORC1 mammalian target of rapamycin complex 1, AMPK adenosine 5′-monophosphate (AMP)-activated protein kinase, ASS1 argininosuccinate synthetase 1, SPAG4 sperm associated antigen 4, SREBP1 sterol responsive element binding protein 1, SCD1 stearoyl-CoA desaturase-1, SCD5 stearoyl-CoA desaturase-5, SFA saturated fatty acids, MUFA monounsaturated fatty acids, BCAT2 Branched-chain amino acid aminotransferase 2, BCAA branched-chain amino acids. BCKA branched-chain α-ketoacids, αKG alpha-ketoglutaric acid, Glu glutamate, FADS2 fatty acid desaturase 2, ZEB1 zinc finger E-box-binding homeobox protein 1, GSH reduced glutathione, GPX4 glutathione peroxidase 4, PKCβII protein kinase C βII, RB1 retinoblastomal 1, PUFA polyunsaturated fatty acids, ACSL4 long-chain acyl-CoA synthetase 4, LPCAT3 lysophosphatidylcholine acyltransferase 3, SOAT1 sterol O-acyltransferase 1, PE phosphatidylethanolamine, CE cholesterol ester, LOXs lipoxygenases, CDK1 cyclin-dependent kinase 1, UBR5 ubiquitin ligase E3 component N-recognition protein 5, Met methionine, SAM S-adenosylmethionine, ACSL3 long-chain acyl-CoA synthetase 3, OA oleic acid (Created with BioRender.com).

Lipids are essential for cells and organisms and are involved in multiple biological processes. According to The LIPID MAPS Lipid Classification System proposed by the NIH, lipids can be divided into several categories, including fatty acyls (FA), glycerolipids (GL), glycerophospholipids (GP), sphingolipids (SP), sterol lipids (ST), prenol lipids (PR), glucosylsphingoshine (SoG1) and polyketides (PK). Lipid metabolism can be regulated in various processes, such as uptake, synthesis, storage and release, which might influence ferroptosis in cancer cells (Fig. 3).

### Phospholipids

Phospholipids are the major components of biomembranes, which are composed of two hydrophobic fatty acyl chains and a hydrophilic head group. Two fatty acyl chains generally consist of saturated fatty acids (SFAs) or monounsaturated fatty acids (MUFAs) in the sn1 position and SFAs, MUFAs or PUFAs in the sn2 position. A great number of evidences suggest that phospholipids play a vital role in initiation and development of ferroptosis [16, 88]. Phospholipid peroxidation can be catalyzed by oxidoreductases, including POR and NADH-cytochrome b5 reductase (CYB5R1), which can generate hydrogen peroxide (H_2_O_2_) through transferring electron and hydrogen atoms from NAD(P)H to oxygen, following which H_2_O_2_ produces reactive hydroxyl radicals through Fenton reaction for the peroxidation of membrane PL-PUFA chains, thus vandalizing the integrity of the cellular membrane and initiating ferroptosis [14].

### Fatty acids

The composition of fatty acids in phospholipids determines the fate of cancer cells. On the basis of saturation of the hydrocarbon chains, fatty acids can be divided into SFAs, MUFAs and PUFAs. Only when the sn2 position of phospholipids is mainly composed of PUFAs, ferroptosis can be initiated by the phospholipids peroxidation [89]. According to the position of the first unsaturated bond, PUFAs can be further classified into omega-3, omega-6, omega-7 and omega-9 PUFAs. However, current studies have suggested that only omega-3 and omega-6 PUFAs participate in the regulation of the progression of malignant neoplasms [90]. The kinds of fatty acids in the sn2 position can be influenced by the ingestion and synthesis of fatty acids, while the de novo synthesis of fatty acids might play a more important role than ingestion in cancer cells [91]. Even so, the effect of dietary ingestion of PUFAs on cancer cells cannot be ignored. Contrary to the traditional concept that increased lipids or PUFAs uptake can sensitize cancer cells to ferroptosis, an increasing number of researchers believe that excessive intake of omega-3 PUFAs might be associated with increased expression of lipogenic enzymes, which can promote the development of cancers [92–94].

#### Fatty acid anabolism

The synthesis of unsaturated fatty acids includes transportation of acetyl-CoA, the synthesis of malonyl-CoA and fully saturated 16 carbon fatty acid (palmitate), elongation and desaturation.

##### Synthesis of SFAs

Acetyl-CoA carboxylase 1 (ACC1), a rate-limiting enzyme in fatty acid synthesis, catalyzes the reaction from acetyl-CoA to malonyl-CoA. ACC1 can be phosphorylated by AMP-activated protein kinase (AMPK), which inhibits its enzymatic activity, thus restraining the generation of fatty acids, particularly PUFAs, finally resulting in insensitivity to ferroptosis [95]. Fatty acid synthase (FASN) is another key enzyme of fatty acid synthesis, which catalyzes the conversion of malonyl-CoA to palmitate. A recent study demonstrated that FASN can be upregulated by mutant KRAS, which increases the synthesis of SFA and MUFA, thus suppressing ferroptosis [96]. FASN can be inhibited by the AMPK/ACC1 pathway [96]. FASN can bind to hypoxia-inducible factor 1-alpha (HIF-1α) to prompt its translocation, suppress its ubiquitination and proteasomal degradation, and then increase the transcription of *SLC7A11*, thus resulting in the cellular confrontation to ferroptosis [97].

##### Elongation

In the initial step of elongation, 3-ketoacyl-CoA synthase acts as a catalyst in the condensation reaction of malonyl-CoA with a fatty acyl-CoA precursor. 3-ketoacyl-CoA synthase can be encoded by seven ELOngation of Very Long fatty acids genes, designated ELOVL1–ELOVL7. Among these seven genes, ELOVL5 and ELOVL6 play important roles in the regulation of lipid metabolism and ferroptosis. High levels of ELOVL5 have been found in mesenchymal-type gastric cancer cells (GCs), where they can increase the generation of arachidonic acid (AA) and adrenic acid (AdA), thus sensitizing tumor cells to ferroptosis [98]. Kagan et al. observed that AA and AdA-containing phosphatidylethanolamine (PE) molecules tend to be oxidized and subsequently induce ferroptosis [99]. Consistent with these insights, Tonevitsky et al. identified that low expression levels of ELOVL5 in breast cancer is correlated with poor prognosis because ELOVL5 can produce PUFAs that can induce ferroptosis in cancer cells [100]. Besides that, a recent study demonstrated that another member of ELOVL family, ELOVL6, can boost ferroptosis through interacting with ACSL4 [101]. However, the underlying mechanism of how ELOVL6 interacts with ACSL4 remains unclear. In the following steps of elongation, 3-ketoacyl-CoA reductase, 3-hydroxyacyl-CoA dehydratase and trans-2,3-enoyl-CoA reductase catalyze reduction, dehydration and reduction, respectively. However, whether these three enzymes regulate cancer development in a ferroptosis-dependent manner requires further exploration.

##### Desaturation

Like elongation, desaturation is also a crucial process in the production of PUFAs. Three fatty acyl-CoA desaturases, including delta-5-eicosatrienoyl-CoA desaturase (D5D), delta-6-oleoyl(linolenoyl)-CoA desaturase (D6D) and delta-9-stearoyl-CoA desaturase (SCD), introduce desaturation in various fatty acids. D5D, which can introduce double bonds at C5 of multiple fatty acids, is encoded by *FADS1* [102]. It has been reported that inhibition of fatty acid desaturase-1 (FADS1) lowers the levels of AA and AdA in gastric cancer, thus resulting in tumor insensitivity to ferroptosis [98]. Stearoyl coenzyme A (CoA) desaturase-1 (SCD1) plays an important role in the desaturation of fatty acids, which catalyzes the formation of MUFAs from SFAs [103]. Sterol responsive element binding protein (SREBP1), a member of the SREBP family, regulates de novo lipogenesis by controlling the transcription of associated genes, such as SCD1 [104, 105]. Sperm associated antigen 4 (SPAG4) promotes the expression and nuclear translocation of SREBP1 through binding to lamin A/C [106]. Activation of the PI3K-AKT-mTOR signaling pathway upregulates the function of SREBP1, thus promoting the transcription of SCD1, which can generate MUFAs to mitigate ferroptosis in cancer cells [104]. Furthermore, AMPK phosphorylation can be induced to suppress the expression of SREBP1, which can translocate to the nucleus to activate the transcription of BCAT2, eventually resulting in ferroptosis [86]. In addition, alpha 1,3-mannosyltransferase (ALG3) and GPX4 can control the expression of SREBP1 to regulate ferroptotic cell death [107, 108]. In addition to SCD1, SCD5 is another isoform of SCDs, which cannot only regulate the ratio of MUFAs and SFAs, but also regulate the synthesis of polar and neutral lipids [109]. In a recent study by Zhang et al., argininosuccinate synthase (ASS1) was shown to actuate the mTORC1-SREBP1-SCD5 axis, which can facilitate the synthesis of MUFAs, thus resulting in ferroptosis resistance [110].

Fatty acid desaturase 2 (FADS2), a key fatty acid desaturase, catalyzes the synthesis of sapienate from palmitate [111]. FADS2, has opposite effects on ferroptosis in various cancers, which is a double-edged sword in the regulation of tumor onset and development. In ascites-derived ovarian cancer cells, FADS2 inhibition disrupts the function of GPX4, which decreases the GSH/GSSG ratio and increases the levels of lipids peroxidation, eventually leading to ferroptosis [94]. In mesenchymal pancreatic cancer cells, the transcription activity of FADS2 can be upregulated by the increased translocation of zinc finger E-box-binding homeobox protein 1(ZEB1) induced by its O-GlcNAcylation, subsequently resulting in the accumulation of lipid peroxidation and ferroptosis [112]. FADS2 expression can be regulated by lymphoid-specific helicase (LSH) or cannabinoid receptor 1 (CB1) [113, 114]. LSH can facilitate the expression of FADS2 and SCD1, which can increase the mRNA levels of ferroptosis-associated genes, such as *SLC7A11* and *GLUD1*, thus decreasing the intracellular levels of Fe^2+^ and lipid ROS, finally suppressing ferroptosis in lung cancer [113]. CB1 can upregulate the expression of SCD1 and FADS2 to promote erastin/RSL3-induced ferroptosis via the AKT and ERK pathways in triple-negative breast cancer (TNBC) cells [114]. Intriguingly, SCD and FADS2 have recently been implicated to involve in the inhibition of ferroptosis and are associated with tumor infiltration of some immune cells, such as dendritic cells (DCs) and B cells, whose expression levels can be used to predict prognosis and disease-free survival [115]. Therefore, Luo et al. identified SCD and FADS2 as potential antigens of mRNA vaccines targeting bladder cancer (BCa) [115]. Coincidentally, some studies have demonstrated that FADS2 is a ferroptosis-related gene (FRG) that can predict overall survival (OS) probability in lung squamous cell carcinoma (LUSC) and BCa, thus providing a novel target for precise therapy [116, 117].

#### Fatty acid catabolism

In cancers, lipid catabolism is as important as anabolism and regulate cancer onset, development and progression. Fatty acid catabolism is an important composition of fatty acid metabolism. Acyl-coenzyme A synthetases (ACSs) participate in the initiation/activation of the fatty acid catabolic pathway, which catalyzes the thioesterification of fatty acids and CoA [118]. The ACS family contains 26 members, which can be roughly divided into short-chain ACS (ACSS), medium-chain ACS (ACSM), long-chain ACS (ACSL), very long-chain ACS (ACSVL), bubblegum ACS (ACSBG) and ACS family (ACSF) [118]. Among these ACSs, ACSLs play a vital role in the initiation and progression of cancers mediated by ferroptosis. The ACSL family includes five members, ACSL1, ACSL3, ACSL4, ACSL5 and ACSL6.

In recent years, many bioinformatics analyses have identified ACSL1 as an indicator of active fatty acid metabolism and ferroptosis, indicating that it can also be a biomarker for predicting the prognosis of cancers and recognizing the phenotype of the tumor environment (TME) [119–121]. Protein arginine methyltransferase 1 (PRMT1) can promote histone methylation in the promoter region of *ACSL1*, thus decreasing the level of lipid peroxidation and mediating ferroptosis resistance in acute myeloid leukemia (AML) [122]. ACSL1 mediates alpha-eleostearic acid (αESA)-induced ferroptosis through increasing the accumulation of hydroperoxide in TNBC cells [123]. Besides, the transcriptional levels of ACSL1 can be regulated by super-enhancers (SEs) formed by bromodomain-containing protein 4 (BRD4) and high mobility group box 2 (HMGB2) [124]. However, in ovarian cancer, Zhang et al. have recently identified ACSL1 as a ferroptosis suppressor that can promote the formation of N-myristoylated FSP1 and subsequently suppress lipid peroxidation [125].

Recent studies have shed light on the role of ACSL3 in ferroptosis. ACSL3 mediates ferroptosis resistance by activating MUFAs [126]. ACSL3 can assist oleic acid in promoting erastin-induced ferroptosis resistance to accelerate tumor metastasis in melanoma cells [127]. Similarly, mammary adipocytes secrete oleic acid dependent on ACSL3 to downregulate the levels of lipid peroxidation and suppress ferroptosis to protect breast cancer cells [128]. Moreover, MAT2A can produce SAM, which trimethylates the promoter of *ACSL3*, leading to ACSL3 upregulation and ferroptosis resistance in gastric cancer [71]. Consistent with these insights, many bioinformatics analyses have reported that ACSL3 can be used to predict the prognosis of various cancers [129–133].

A growing body of evidences suggest that ACSL4 plays a key role in ferroptosis. ACSL4 was first identified as a navigator of ferroptosis by haploid genetic screening [13]. The main function of ACSL4 in ferroptosis initiation is to catalyze the formation of PUFA-CoA, which can be utilized to produce PUFA-PE or PUFA-CE by lysophosphatidylcholine acyltransferase 3 (LPCAT3) or sterol O-acyltransferase 1 (SOAT1), thus promoting lipid peroxidation and ferroptosis [134]. AA is the most abundant and widespread ω-6 PUFA in the human body. Previous studies have found that ACSL4 catalyzes the activation of AA to AA-CoA, which can be utilized to generate 5-hydroxyeicosatetraenoic acid (5-HETE) by lipoxygenase (LOX), thus inducing ferroptosis via 5-HETE-mediated lipotoxicity [135]. Besides, ACSL4 sensitizes tumor cells to ferroptosis through increasing the proportion of AA-containing PE species in the cellular membrane [12]. AA-PE can be oxidized to AA-PE-POOH, whose accumulation can induce ferroptotic cell death [99]. Protein kinase C βII (PKCβII), a lipid peroxidation sensor, can promote ACSL4 to catalyze the production of PUFAs and increase the levels of lipid peroxidation, finally inducing ferroptosis [136]. These effects can be strengthened by interferon gamma (IFNγ) secreted by CD8^+^ T cells [137]. Therefore, a promising strategy that combines immune checkpoint inhibitors (ICIs) with ferroptosis inducers that activate the PKCβII-ACSL4 pathway can be introduced in cancer therapy. The upstream molecular of ACSL4, cyclin-dependent kinase 1 (CDK1) can mediate ubiquitin-mediated degradation of ACSL4 via phosphorylating ACSL4 and recruiting ubiquitin ligase E3 component N-recognition protein 5 (UBR5), ultimately causing ferroptosis and chemotherapy resistance in CRC [138]. RB1 is another upstream suppressor of ACSL4, whose loss can activate transcription factor E2F1 binding to the promoter of ACSL4 by increasing the transcriptional levels of *E2F1*, thus promoting the occurrence of ferroptosis [139]. These findings provide a novel therapeutic target for *RB1*-deficient cancers.

ACSL5 has been reported to promote the generation of ROS and the accumulation of lipid peroxidation, ultimately sensitizing tumor cells to ferroptosis [140].

In addition to the ACSL family, *ACSF2* has been reported to be a ferroptosis-related gene, which is correlated with poor prognosis in cancer, while the specific mechanism of ACSF2 on ferroptosis-related cancers remains unclear [141–143]. Malonyl-CoA decarboxylase (MLYCD) plays an important role in fatty acid catabolism, which catalyzes the conversion of malonyl-CoA to acetyl-CoA. Chen et al. identified MLYCD as a regulator of ferroptosis sensitivity in clear cell renal cell carcinoma (ccRCC) and identified a novel therapeutic target for ccRCC [144]. MLYCD can suppress the activity of CPT1A and increase the levels of PUFAs, ROS and malondialdehyde (MDA), an indicator of ferroptosis. In addition, overexpression of MLYCD can inhibit the expression of ferroptosis-negative genes, such as *SCD1*, and upregulate ferroptosis-positive genes, including *PTGS2*, *ALB* and *AQP3*.

### Cholesterol

**Fig. 4:**
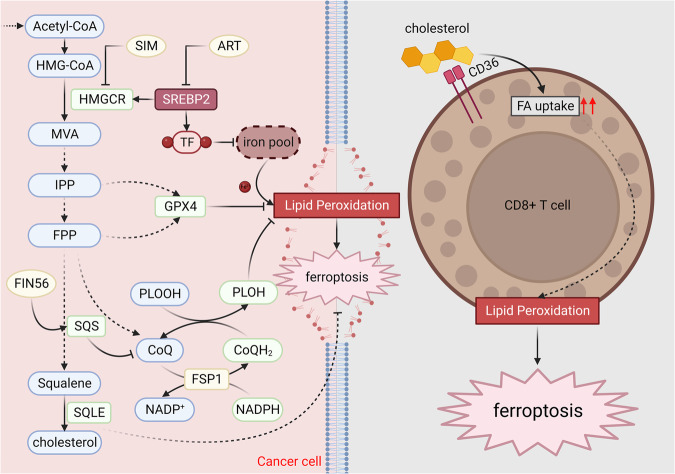
Ferroptosis and cholesterol metabolism. Cholesterol synthesis is catalyzed by a series of enzymes, such as HMGCR, SQS and SQLE, via MVA pathway. SREBP2 regulates the expression of HMGCR and the levels of TF, which inhibits lipid peroxidation and ferroptosis. MVA pathway can regulate the synthesis of GPX4 and CoQ, which are the negative regulators in ferroptosis. Besides, cholesterol in the tumor microenvironment can increase the expression of CD36 in tumor-infiltrating CD8^+^ T cells, which can increase FA uptake and promote lipid peroxidation, thus inducing ferroptosis in CD8^+^ T cells and maintaining the survival of cancer cells. HMG-CoA 3-hydroxy-3-methylglutaryl-CoA, HMGCR HMG-CoA reductase, MVA mevalonic acid, IPP isopentenyl pyrophosphate, FPP farnesyl pyrophosphate, SQS squalene synthase, FIN56 ferroptosis inducer 56, SQLE squalene epoxidase, SIM simvastatin, ART artesunate, SREBP2 sterol responsive element binding protein 2, TF transferrin, GPX4 glutathione peroxidase 4, PLOOH phospholipid hydroperoxides, PLOH phospholipid alcohols, CoQ ubiquinone, CoQH_2_ ubiquinol, NADPH reduced nicotinamide adenine dinucleotide phosphate (Created with BioRender.com).

Cholesterol, a kind of sterol lipid, is an important component of mammalian membranes, which plays a key role in maintaining membrane integrity and fluidity. Cholesterol metabolism includes biosynthesis, uptake, efflux, and esterification. Cholesterol is synthesized through the mevalonate pathway, which contains a series of complex reactions (Fig. 4).

In these processes, acetyl-CoA is the initial molecule that can be used to generate 3-hydroxy-3-methylglutaryl-CoA (HMG-CoA). HMG-CoA can be reduced to mevalonic acid (MVA) by HMG-CoA reductase (HMGCR), which is the first rate-limiting enzyme in cholesterol biosynthesis. Inhibition of HMGCR by simvastatin (SIM) can indirectly suppress the mevalonate pathway and GPX4 expression, thus promoting ferroptosis in TNBC [145]. However, the direct mechanism of HMGCR on cancer progression dependent of ferroptosis remains unclear. SREBP2, another member of the SREBP family, is a key regulator of cholesterol synthesis. Its nuclear translocation can be inhibited by the antimalarial drug artesunate (ART), which leads to decreased transcriptional levels of *HMGCR* and reduced levels of IPP, thus impeding the synthesis of GPX4 and resulting in ferroptosis [146]. Besides, SREBP2 can induce the expression of transferrin (TF), which is an iron-sequestration protein that can decrease the intracellular iron pool, thus suppressing lipid peroxidation and ferroptosis [147].

MVA can be phosphorylated and decarboxylated to generate isopentenyl pyrophosphate (IPP). IPP can be utilized to synthesize GPX4 and ubiquinone (oxidized CoQ). IPP participates in the isopentenylation of selenocysteine tRNA (Sec-tRNA), which is essential for the transportation of selenocysteine to synthesize GPX4 [148]. Moreover, IPP can be used to synthesize ubiquinone, which can be reduced to ubiquinol (reduced CoQ) by FSP1, subsequently trapping lipophilic radicals that mediate lipid peroxidation and ultimately impeding ferroptosis [16, 149]. Therefore, inhibition of IPP can sensitize cancer cells to ferroptosis in a GPX4/FSP1-dependent manner. Farnesyl pyrophosphate (FPP) is the downstream molecule of IPP, which also participates in the generation of ubiquinone. The mechanism of FPP on ferroptosis is the same as IPP.

The conversion of FPP to squalene is catalyzed by squalene synthase (SQS). A recent study demonstrated that squalene, an active component of fermented soybean lipids (FSE-C), might suppress ferroptosis through increasing the level of GSH, decreasing the level of Fe^2+^ and regulating the transcription levels of FRGs, such as *SLC7A11* and *ACSL4*, in rat pheochromocytoma cells [150]. Activation of SQS by FIN56 decreases the synthesis of ubiquinone and contributes to the accumulation of lipophilic radicals that can induce ferroptosis [151]. Squalene epoxidase (SQLE) catalyzes the oxidation of squalene to 2,3-epoxysqualene, which can eventually be transformed into cholesterol. SQLE mediates ferroptosis resistance in breast cancer via decreasing the ubiquitination of CCNB1 and the intracellular levels of ROS [152].

Interestingly, 27-hydroxycholesterol (27HC), a cholesterol metabolite, can increase cellular lipid uptake and/or biosynthesis, thus increasing intracellular metabolic stress, which can upregulate the expression of GPX4 and suppress ferroptosis, ultimately promoting tumorigenicity and metastasis [153]. Meanwhile, cholesterol can be used to form lipid rafts and decrease the fluidity of the cellular membrane, which can inhibit the diffusion of lipid peroxidation and the occurrence of ferroptosis, thus promoting the survival of cancer cells in the TME [154]. In addition, cholesterol in the tumor environment can upregulate the expression of CD36 in tumor-infiltrating CD8^+^ T cells, which can increase the ingestion of fatty acids, promote lipid peroxidation and ferroptosis as well, thus compromising the anti-tumor function of T cells and promoting tumor progression [155]. Low-density lipoprotein receptor (LDLR) is essential for maintaining cholesterol homeostasis, which can sensitize cancer cells to ferroptosis through inhibition of GPX4 via mediating cholesterol uptake [154]. Furthermore, a recent study by Zhong et al. demonstrated that inhibition of LDLR can promote ferroptosis through the PI3K/AKT pathway in diffuse large B-cell lymphoma (DLBCL) [156].

## Conclusion

Ferroptosis is a non-apoptotic cell death that is triggered by lipid peroxidation in the presence of iron. Cancer cell metabolism is mainly composed of carbohydrate metabolism, amino acid metabolism and lipid metabolism, in which enzymes and substrates can regulate ferroptosis through multiple mechanisms. These enzymes and substrates can be explored as therapeutic targets for cancer. For example, NADPH produced during carbohydrate metabolism and the raw materials of GSH generated during amino acid metabolism suppress ferroptosis, whereas PUFAs produced during lipid metabolism can trigger ferroptosis.

Notably, ferroptosis-targeted therapy can be combined with immunotherapy to improve the treatment of cancer. Even so, many regulatory mechanisms still need to be explored and clarified: (1) Which molecule acts as an executor in ferroptosis? (2) Why do some molecules play opposite roles in ferroptosis, and what is the underlying mechanisms? (3) With the exception of lipid peroxidation, are there any other markers for ferroptosis detection? Taken together, these data show that the interaction between ferroptosis and cancer metabolism needs to be studied further for increased clarity.
